# eWoT: A Semantic Interoperability Approach for Heterogeneous IoT Ecosystems Based on the Web of Things

**DOI:** 10.3390/s20030822

**Published:** 2020-02-04

**Authors:** Andrea Cimmino, María Poveda-Villalón, Raúl García-Castro

**Affiliations:** Ontology Engineering Group (OEG), Universidad Politécnica de Madrid, 28660 Madrid, Spain; rgarcia@fi.upm.es

**Keywords:** semantic interoperability, IoT devices, context-based search, content-based search

## Abstract

With the constant growth of Internet of Things (IoT) ecosystems, allowing them to interact transparently has become a major issue for both the research and the software development communities. In this paper we propose a novel approach that builds semantically interoperable ecosystems of IoT devices. The approach provides a SPARQL query-based mechanism to transparently discover and access IoT devices that publish heterogeneous data. The approach was evaluated in order to prove that it provides complete and correct answers without affecting the response time and that it scales linearly in large ecosystems.

## 1. Introduction

In the last decade, the IoT devices available through the Web have become pervasive [1], allowing users and applications to easily monitor and interact with their devices [2]. Far from reaching a scenario in which the number of IoT devices will stop growing, researchers foresee a constant growth in the number of such devices available through the Web in the mid-term future [3,4]. The different IoT devices are developed and distributed by different vendors [5]; as a result, these IoT devices rely on a wide number of heterogeneous models, data formats, and APIs [6].

Semantic interoperability aims at dealing with IoT device heterogeneity [7], by enabling an environment in which IoT devices can be included, forming an IoT ecosystem. The ecosystem must enable transparent mechanisms through which such devices are discoverable [8]. Discovery should enable context- and content-based searching (i.e., the discovery of sensors by some meta-data (context-based) or by values that devices are capturing (content-based)). For this purpose, W3C standards have become widely adopted [9], specifically SPARQL [10], to transparently interact with IoT ecosystems, and ontologies are used to describe the meta-data of the IoT devices. In particular, the Thing Description (TD) model defined by the W3C Web of Things working group [11] is one of the most adopted to profile IoT devices and developing discovery mechanisms [12].

A thorough analysis of the current proposals that address semantic interoperability for IoT ecosystems was presented by Zout et al. [9], involving approximately 50 proposals. There are mainly two approaches for Web-available IoT devices based on the W3C standards that perform both context- and content-based searches: federation and centralised approaches. The former requires each IoT device in the ecosystem to enable a SPARQL endpoint, so when a query is issued it is federated to all the devices in the ecosystem [13]. The latter consists of storing all the sensors’ meta-data in a central triple store, and it requires the IoT devices to push their fresher data into the triple store. The federation approaches do not deal with the IoT heterogeneity; they rely on the restriction that IoT devices manage SPARQL queries, which considering the heterogeneity nature of such devices is not likely to occur. The centralised approaches deal with the heterogeneity of the devices by requiring them to periodically send data, which compromises the freshness of the information and requires practitioners to implement synchronization mechanisms.

In this article, we introduce a novel approach called eWoT that allows transparent interaction by means of SPARQL queries with an IoT ecosystem that is formed by Web-available IoT devices. The approach relies on Thing Descriptions (TDs) to profile the different IoT devices [11]. Unfortunately, these TDs are not expressive enough to deal with the heterogeneity of the IoT devices [14,15]. For this reason, this article presents an extension of Thing Descriptions called WoT-Mappings that specify how data from IoT devices can be translated to RDF (Resource Description Framework) on the fly, thus dealing with their heterogeneity. Relying on both the Thing Descriptions and the WoT-Mappings that are stored in the triple store, eWoT is able to perform a discovery based on both context-based and content-based search when a SPARQL query is issued.

The novelty of our approach relies on two main pillars: (a) The WoT-Mappings that extend the Thing Description model, allowing to define IoT ecosystems that are profiled by the Thing Descriptions and whose data can be translated into RDF thanks to the WoT-mappings. (b) The Thing Descriptions and the WoT-mappings are allocated in a centralised triple store, but when a SPARQL query is issued, eWoT performs a distributed access to the IoT devices and translates their data on the fly. As a result, our approach deals with the heterogeneity of IoT devices non-intrusively and does not require IoT devices to manage SPARQL or to send any data. In addition, our proposal is fully based on standards.

eWoT was validated in three experiments. The goal of the first was to prove that the query answers produced by eWoT are complete, correct, and that eWoT is as efficient as a triple store when performing a context-based search. The second experiment aimed at studying the scalability of eWoT and at analysing the overhead that the content-based search introduces due to real-time distributed access and data translation on the fly. The third experiment aimed at comparing eWoT with a proposal that implements a different semantic interoperability approach from the literature, analysing their pros and cons. The results advocate that a query could be answered in the best case in 6 s involving 100 IoT devices, and in the worst case in approximately 75 s when 1,000 IoT devices were involved. In all cases, the devices must be discovered, their data accessed, translated into RDF, combined with their descriptions, and finally the query answer is computed.

The rest of this article is structured as follows: Section 2 introduces an analysis of some proposals from the literature; Section 3 and Section 4 report our main contributions (i.e., the WoT-Mappings and the eWoT semantic interoperability approach); Section 5 explains the experiments carried out to validate eWoT, as well as the results obtained and, finally, Section 6 recaps our findings and conclusions.

## 2. Related Work

There are many proposals addressing the challenges associated with semantic interoperability. However, it is important to distinguish between: (a) those proposals that implement semantic interoperability for just one specific IoT device [16,17,18,19,20,21,22,23,24,25,26,27,28], and (b) those that aim at implementing a semantically interoperable IoT ecosystem in which transparent mechanisms to interact with the IoT devices within are provided [8,29,30,31,32,33,34,35].

The semantic interoperability approaches that provide mechanisms to transparently interact with a single IoT device usually consist of specifying a common API, data format, and data model. Then, the IoT devices must adapt to such specifications by either developing adapters or using some kind of service-wrapper. The proposals that implement semantic interoperability for just one IoT device are out of scope for this paper, since the main problem addressed in this paper consists of the broader problem of enabling transparent mechanisms to interact with the IoT devices within an IoT ecosystem.

Zhou et al. [9] analysed a large number of proposals from the literature that implement semantic interoperability for IoT ecosystems. As a result, they provided a classification for the different approaches used by the proposals to enable transparent mechanisms to interact with the IoT ecosystem. These are basically to discover devices using a search criteria (context-based search) and to access them in order to retrieve their data and include such information as output for the search criteria (content-based search). The proposals are grouped into these categories: Indexing, Clustering, Linked Data, Streaming Data, O&M Data, Sensor Information, and Entity Information. From all these categories we focus on the Linked Data, which has two main sub-categories (i.e., centralised and federation approaches).

***Centralised approaches*** have a good efficiency, although they require the data from the IoT devices to be centralised in a triple store, introducing a number of limitations such as scalability, how data is pushed by the IoT devices (and thus translated into RDF), and the freshness of pushed data when solving a SPARQL query [9].

eWoT differs from centralised approaches in that it does not store IoT device data into a central repository, but only centrally stores WoT Thing Descriptions that profile IoT devices. Therefore, it performs a decentralised distributed access to those devices that are relevant to answer the issued query, and translates their data on the fly in order to perform both context-based (with the Thing Descriptions) and content-based search (thanks to the WoT-Mappings).

***Federation approaches*** assume that the IoT devices of the ecosystem have a SPARQL endpoint enabled. They exploit this assumption by treating each device as a distributed triple store that can be queried by means of federation techniques to answer a SPARQL query [13].

Our proposal differs from the federation approaches since they require IoT devices in the ecosystems to enable a SPARQL endpoint, whereas eWoT deals with IoT devices that rely on heterogeneous APIs, formats, and models; therefore, eWoT is less restrictive. As a result, eWoT answers a SPARQL query considering both context- and content-based search as the federation approaches do.

Table 1 summarizes the proposals that we have identified in the literature according to the specifications of Zhou et al. [9].

To our best knowledge, eWoT is the first proposal to perform this kind of search. Therefore, we have extended the classification of Zhou et al. [9] including a new category called *decentralised*. This approach consists of performing a context-based search over a set of Thing Descriptions, and then enhancing these descriptions with data retrieved from relevant devices and performing a decentralised access on the fly, enabling a content-based search. Our approach has the benefits of the centralised approaches, plus the scalability of the federation approaches. In addition, our proposal always guarantees the freshness of the data.

In this article we introduce a novel approach that allows building semantically interoperable ecosystems of IoT devices. Our approach offers a SPARQL-query-based mechanism to transparently discover and access devices in the ecosystem, and to produce an answer even if the devices do not rely on RDF or on semantic Web technologies.

Our approach relies on two contributions: (a) the WoT-Mappings ontology that enables the description of how to translate the data from the heterogeneous IoT devices that form the interoperable ecosystem, and (b) the eWoT system that registers the IoT devices into the ecosystem and enables a SPARQL-based transparent mechanism to discover and access them.

## 3. Semantic Specification of Thing Ecosystem Descriptions

**Figure 1:**
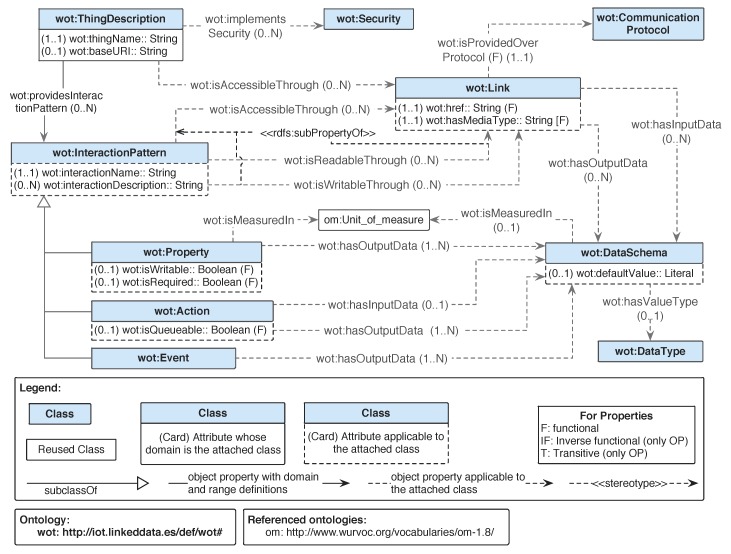
Graphical description of the Web of Things (WoT) ontology.

Our approach builds IoT ecosystems by gathering WoT Thing Descriptions (TDs) of IoT devices. The Thing Description is an RDF document that specifies the interaction patterns that a device may have (i.e., properties, actions, and events). The interactions describe information regarding the endpoints, that is, the URL, whether they are readable/writable, or the media type of the data. In order to rely on the WoT Thing Descriptions, Figure 1 presents the ontology that we have developed, which is later explained in detail in Section 3.1.

It has been proven that these descriptions can be used to perform a transparent context-based discovery among all the described IoT devices for a given SPARQL query [9], and can be extended with contextual data to enhance such discovery (e.g., with locations). Nevertheless, the Thing Descriptions are not expressive enough to allow transparent access to the data of the IoT devices. This is due to the heterogeneity of their data formats and models and because they do not describe how to interpret such heterogeneous data.

**Figure 2:**
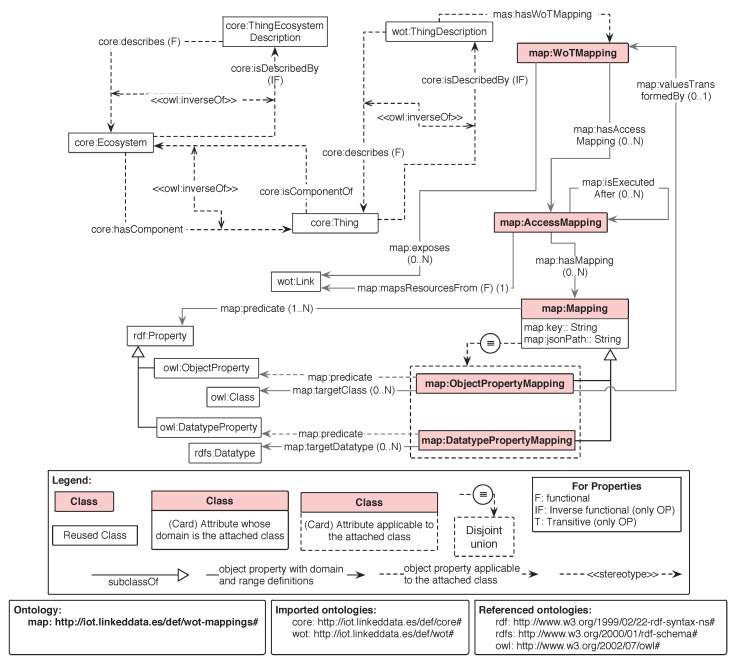
General overview of the WoT-Mapping ontology.

The heterogeneity in the formats and models can be addressed by translating such data into RDF expressed with an ontology, and injecting such information in the Thing Description. The mappings are RDF documents that describe how to perform such translation on the fly [36]. Note that these mappings could also be used to translate an RDF document expressed with one ontology into another expressed with another. Our approach extends the Thing Description by including mappings modelled by the WoT-Mappings ontology, which is depicted in Figure 2 and detailed later in Section 3.2.

The Thing Descriptions shown in Figure 1 together with the functionality of the WoT-Mappings shown in Figure 2 allow an an IoT ecosystem to be transparently described. For this reason, a new concept has been introduced, that is, the Thing Ecosystem Description (TED). As can be seen in Figure 2, the TED describes an interoperable ecosystem by means of a set of Thing Descriptions (one per IoT device), which may include a set of mappings. The Thing Ecosystem Description can be extended with new Thing Descriptions anytime a new IoT device must be integrated in such an ecosystem, becoming in this way interoperable.

Ontologies allow the generation of semantic descriptions and the annotation of different entities in the IoT domain, including the description of any type of entity (physical or virtual) as well as their Web representations and associated properties, actions, and events. As there is a broad variety in the type of information to be modelled by ontologies, especially in an IoT environment, it is good practice to follow a modular approach for the ontology development and implementation. Under the umbrella of these good practices the WoT and the WoT-Mapping ontologies have been developed, and will be explained in detail in the following sub-sections.

### 3.1. WoT Thing Description Ontology

The WoT ontology was developed to define “what”, “where”, and “how” things within an ecosystem of IoT devices can be discovered or accessed in the Web of Things. It is worth noting that the presented WoT ontology was developed following the definitions and discussions taking place within the W3C WoT Working Group (https://www.w3.org/WoT/WG/) when developing the Thing Description model [11]. Furthermore, our ontology (http://vicinity.iot.linkeddata.es/vicinity/) was the seed for the model being developed in this working group (W3C TD). The main differences between our ontology and the model are: (a) naming differences as wot:InteractionPattern vs. td:InteractionAffordance or wot:Property vs. td:PropertyAffordance; (b) the W3C TD model data schema is defined for JSON while in WoT model the concept wot:DataSchema is defined in a general level agnostic to the technology, and is specialised for JSON in the VICINITY Datatypes ontology; (c) W3C TD includes concepts for versioning and multilingualism while WoT ontology includes does not but includes communication protocol information. Note that the core models are compatible and that the differences are a product of the parallel developments since the first version of the proposed WoT ontology, which will be aligned with the final W3C TD model when this work is published as a recommendation.

The main concepts defined in the WoT ontology, as shown in Figure 1, are [15]:

- wot:ThingDescription: represents anything, physical or virtual, which has a distinct and independent existence and can have one or more Web representations.
- wot:InteractionPattern: represents, in the context of WoT, an exchange of data between a Web client and a Thing, that is, it specifies how data can be accessed.
- wot:Endpoint: represents the Web location where a service can be accessed by an application, that is, from where the data can be fetched.
- wot:DataSchema: represents the input data or output data of a given interaction pattern, including information such as the data type used and which unit of measurement the data is represented in, if needed. It contains an excerpt of the model.

The WoT ontology allows linking particular things to the interaction patterns it provides by means of the object property wot:providesInteractionPattern. Three types of interaction patterns are defined, such that an interaction pattern can be either a *Property*, an *Action*, or an *Event*.

The model allows *Thing Descriptions* or interaction patterns to be associated to one or more endpoints by means of the object property wot:isAccessibleThrough. The endpoints indicate the Web location in which the service is provided through the datatype property wot:href and this value should be unique for a given endpoint.

### 3.2. WoT-Mapping Ontology

The goal of the WoT-Mapping ontology is to support the translation of data to RDF, specifying the protocols needed to fetch data and the endpoints where such data can be fetched. The current WoT-Mapping ontology is depicted in Figure 2. Other parts of the ontology network (e.g., concepts defined in the VICINITY core or wot ontologies) are represented in the figure in order to show the connection between modules, as it represents how the mappings are expected to be used in the overall picture. Notice that the wot:ThingDescription is the concept that has relation with the map:WoTMapping.

The concept map:Mapping allows the connection between a key (map:key) provided within structured data in an on-line resource to the RDF property to which it should be mapped (map:predicate).

The mappings defined are further classified as map:ObjectPropertyMapping or map:DatatypePropertyMapping (but only one of them) if the predicate linked to them is an owl:ObjectProperty or an owl:DatatypeProperty, respectively.

Another difference between map:ObjectPropertyMapping and map:DatatypePropertyMapping is the target element expected for the transformed values. For a map:ObjectPropertyMapping, the expected target should be an instance of owl:Class by means of the property map:targetClass. For the map:DatatypePropertyMapping, the expected target should be an instance of the class rdfs:Datatype by means of the property map:targetDatatype.

In order to group one or more mappings that are executed over a given endpoint, the map:AccessMapping has been defined. This model allows the definition of mappings independently of the endpoint where they would be executed, as the relation with the endpoint is indicated in the map:AccessMapping instance rather than in the map:Mapping one. Finally, a map:WoTMapping has one or more map:AccessMapping.

Finally, this ontology specifies how one core:ThingEcosystemDescription describes an ecosystem of IoT devices (core:Ecosystem) which contains one or more IoT devices (core:Thing). These devices are profiled by several wot:ThingDescription that may specify how to interact with them by means of the wot:InteractionPattern or how interpret their data by means of wot:WoTMapping.

## 4. Semantic Interoperability Based on the Web of Things

**Figure 3:**
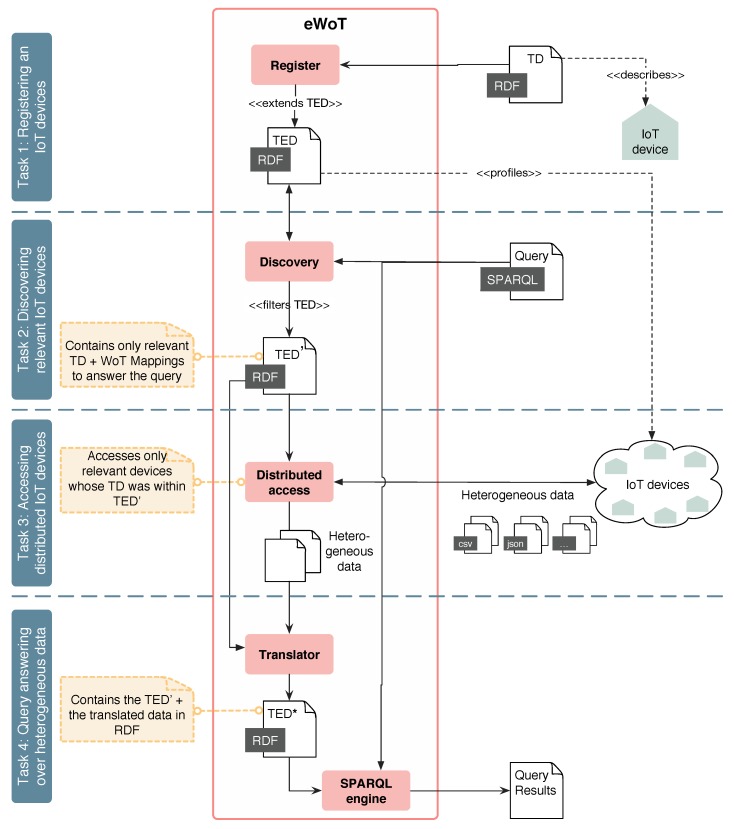
eWoT architecture overview. RDF: Resource Description Framework; TD: Thing Description; TED: Thing Ecosystem Description.

The semantic interoperability that this article presents is built upon the Thing Ecosystem Description. The approach (i.e., eWoT) allows the registration of new IoT devices, and enables a transparent SPARQL-based mechanism to interact with the devices that form the ecosystem. Figure 3 presents the overview of eWoT, which performs the following tasks:

**Task 1: Registering an IoT device.** In order to make a new IoT device compatible within an interoperable ecosystem, a practitioner must provide a Thing Description containing WoT-Mappings in order to extend the TED. In the case that a practitioner provides a Thing Description without mappings, the IoT device will be discoverable but not accessible. Figure 3 depicts how the eWoT extends the TED with the new Thing Description by means of the *Registration* sub-component.

**Task 2: Discovering relevant IoT devices.** When a SPARQL query is issued, eWoT relies on the *Discovery* sub-component that takes the SPARQL query as input and performs a discovery task over the TED. As result, a filtered version of the Thing Ecosystem Description containing only the Thing Descriptions that are relevant to answer the SPARQL query is produced, as reported in Algorithm 1. If a privacy policy should be taken into consideration, it can be applied during this step.

Algorithm 1 reports the discovery algorithm used by the *Discovery* sub-component. It receives as input a SPARQL query (*Q*) and a Thing Ecosystem Description (TED) and returns a narrowed version of the Thing Ecosystem Description ($TED^{\prime }$) containing only relevant Thing Descriptions to answer the query.
**Algorithm 1:** Discovery.
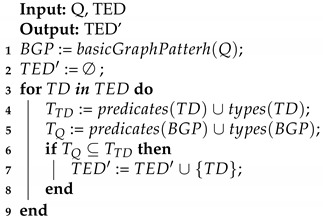


Algorithm 1 first extracts the BGP (the basic graph pattern) of the input query [37], which contains the relevant triples issued in the query. Then, the algorithm iterates over all the Thing Descriptions that are contained in the input TED, which are the Thing Descriptions (TD) of the IoT devices registered. Then, for a Thing Description the predicates and types contained within are extracted by means of the functions *predicates(TD)* and *types(TD)*; these functions can be implemented with one SPARQL query, respectively. The same operation is performed for the triples within the variable BGP. As a result, the sets $T_{TD}$ and $T_{Q}$ containing the list of predicates and types specified in the TD and the BGP are respectively obtained. Finally, the algorithm checks whether all the predicates and types in the BGP are contained in the list of predicates and types of the TD (i.e., $T_{Q}$⊆$T_{TD}$). If that is the case, the Thing Description *(TD)* is included in the filtered version of the TED $\left(TED^{\prime }\right)$.

**Task 3: Accessing distributed IoT devices.** Having a suitable TED that contains only the Thing Descriptions of the relevant IoT devices for answering the issued SPARQL query, eWoT relies on the sub-component Distributed Access to analyse the different Thing Descriptions and fetch their data. The approach considers that the retrieved data is expressed with heterogeneous formats and models, entailing that the IoT devices do not necessarily need to rely on semantic Web technologies. Distributed Access accesses the endpoints described in the TD, and retrieves their heterogeneous data without interpreting it.

**Task 4: Query answering.** Once the heterogeneous data is fetched, the sub-component *Translator* translates the data into RDF by means of the WoT-Mappings associated to the Thing Descriptions used previously to fetch it. As a result, the *Translator* combines the narrowed version of the TED with the translated data, building as a result an RDF document that contains the Thing Descriptions and the RDF generated as result of translating the IoT devices’ data. In Figure 3 this enhanced version of the filtered TED is denoted as TED*.

Finally, the TED* and the issued SPARQL query are provided as input to the sub-component *SPARQL Engine* that solves the query over the RDF data of the TED*. This task is well-known and there are already standard proceedings to solve SPARQL queries over an RDF document [10].

### Implementation

The architecture presented in Figure 3 displays a high-level overview of our implementation. Nevertheless, at the implementation level, we relied on several existing software components and technologies, namely:

- **Triple store:** we relied on the well-known triple store *GraphDB* (https://www.ontotext.com). This repository can be managed as a REST API, which perfectly suits the *Registering Module* sub-component to store the TED and the *Discovery* sub-component to interact with it.
- **Discovery:** we created a Java class that implements the discovery process presented by Algorithm 1.
- **Distributed Access and Translator:** in order to implement the functionalities of these two components, we relied on the Java library Helio (https://helio.linkeddata.es/), which is an engine to generate RDF from heterogeneous data sources (i.e., it performs the distributed access and the translation). We extended the code of this software artefact so it could understand the WoT-Mappings.
- **Register Module and SPARQL Engine:** these components are implemented using Jena 3.6.0. This Java library offers the functionality that answers a SPARQL query for a given RDF document, among other suitable functionalities to handle RDF documents.

eWoT is a Web server that orchestrates all these components and implements the overall functionality. It was implemented with Spring 2.1.5.

## 5. Evaluation

**Figure 4:**
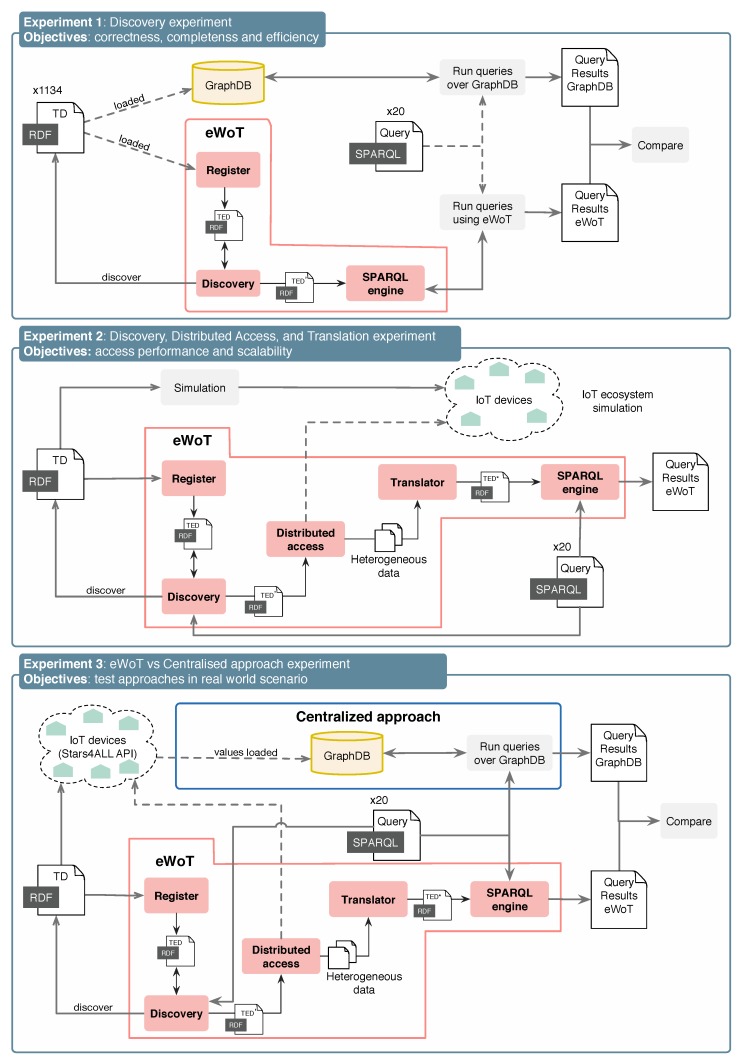
Overview of experimental set ups and processes.

eWoT was evaluated by performing three experiments, each one aiming at validating different features related to its effectiveness and efficiency. Figure 4 depicts an overview of each of them, as well as their setups and the objectives.

- **Experiment 1:** 1134 Thing Descriptions where registered in eWoT without their corresponding WoT-Mappings, restricting the discovery to only context-based search. At the same time, the very same Thing Descriptions were inserted in an external GraphDB triple store. Then, a set of 20 queries were issued to both eWoT and the external GraphDB.
  On the one hand, this experiment aimed at validating whether the query answer produced by eWoT and GraphDB were the same, meaning that the queries answered by eWoT are complete and correct. On the other hand, the response times of eWoT and GraphDB were compared to ensure there were no statistically significant differences between them, meaning that eWoT is as efficient as a triple store when answering queries.
- **Experiment 2:** an incremental number of IoT devices were registered in eWoT up to 1000; for each, a set of 20 queries were issued. This experiment relied on a simulator that creates a variable number of digital twins for smart houses (i.e., IoT devices) that on the one hand register their TD and WoT-Mappings in eWoT and, on the other hand, enable a REST API that publishes JSON data.
  On the one hand, the scalability of eWoT was statistically analysed by simulating 100, 250, 500, 750, and 1000 smart houses. On the other hand, assuming there is no overhead in the transmission between eWoT and the IoT devices, the time that requires the distributed access and the translation of data was analysed in order to verify the amount of overhead that both introduce.
- **Experiment 3:** an incremental number of real-world IoT devices (i.e., photometer data published by the European project Stars4All (https://stars4all.eu/)) were registered in eWoT up to 400; for each, a set of 20 queries was issued. This experiment aimed at comparing the eWoT proposal with a custom centralised approach. The centralised approach consisted of a triple store (i.e., a GraphDB) that stored the TDs of the photometers and a developed custom service that read their values, translated them into RDF, and injected them in GraphDB.
  **Environment:** all the experiments where run in an Ubuntu GNU/Linux x86_64 with four cores and 34 GB of RAM. In this computer, we also simulated all the RESTful endpoints to reduce the network impact when measuring the time taken by the different data exchanges in our experiments. The simulator was implemented using Java 1.8, Spring Boot 2.1.5. For the translation of JSON documents into RDF we used the Helio library (https://helio.linkeddata.es/). To implement our Repository we relied on a GraphDB 8.7.2.
  **Availability:** the implementation of eWoT is publicly available at https://github.com/oeg-upm/eWoT. The simulator used in the experiments, and its manual, can be found under the folder *MDPI-experiments* in the very same repository, where it is specified how to reproduce Experiment 2. Unfortunately, due to confidentiality, the Thing Descriptions involved in Experiment 1 cannot be disclosed publicly. The results obtained in our experiments are publicly available at https://zenodo.org/record/3634897 as a Zenodo repository.
  The query-answering time for both approaches was evaluated by registering 100, 200, 300, and 400 photometers. The scalability in a real-world scenario was analysed for eWoT. In addition, a comparison between both proposals was performed, analysing their pros and cons.

### 5.1. Experiment 1: Discovery

This experiment aimed at testing the *Discovery* component of eWoT, which implements the functionality reported by Algorithm 1. The goal of this experiment was to validate that eWoT produces complete and correct query answers, and is as efficient as a triple store when answering a discovery query that only performs a context-based search.

For this purpose, a set of 20 different queries with different shapes [38] were developed, namely: four linear queries, four star queries, four tree queries, two cycle queries, and six complex queries. The queries with the same shape had an incremental size. For example, the first linear query had four triples whereas the fourth had eight triples. The experiment consisted of running all the queries, ten times each, over GraphDB and over the SPARQL endpoint of eWoT. Every time a query was issued the response time and the content of the query answer were stored. Bear in mind that eWoT computes a TED of suitable IoT devices before answering the query, and therefore introduces additional operations over the query-answering process.

To carry out the experiment, 1134 Thing Descriptions related to IoT devices from the European project VICINITY were loaded into a GraphDB (previously empty) and eWoT, as depicted by Figure 4. These Thing Descriptions profile a wide range of real-world heterogeneous IoT devices. Note that the Thing Description contained no WoT-Mappings, as this experiment aimed at testing only the discovery. Due to confidentiality policies, the Thing Descriptions involved cannot be disclosed publicly.

Table 2 displays the results of this experiment. The column Query contains the shapes of queries issued and the other two columns (i.e., GraphDB and eWoT) recap the results obtained. The sub-column Answer size reports the number of lines contained in the CSV query answer, and the sub-column Avg. Time (s) reports the average query-answering time in seconds.

To ensure the correctness and completeness of the eWoT *Discovery* the query answers produced for the same query by either eWoT and GraphDB were compared. If the size (the number of lines in this case) of the two answers was the same, then eWoT could be considered complete. When the content of both answers was the same, then eWoT could be considered correct. Bear in mind that the query answers in SPARQL hold no order, and thus two answers for the same query may expose the same data sorted differently. In addition, this claim assumes that GraphDB produces complete and correct query answers.

**Completeness:** the completeness was validated by manually comparing the number of lines of all the query answers, and verifying that it was the same for those answers produced by eWoT and those by GraphDB. Although this task was manually verified, the sizes of the query answers are included in Table 2 in order to provide the reader with an idea of the query answer sizes.

**Correctness:** the correctness was validated by manually comparing the content of all the query answers, and verifying that it was the same for those generated by eWoT and by GraphDB. Since the correctness is related with the content of the query answer, there was no suitable way to reflect this in Table 2.

**Discovery Efficiency:**Table 2 reports the average query-answering times (sub-column Avg. Time (s)) for GraphDB and eWoT. The best effort was made to avoid query cache mechanisms in GraphDB. In the light of these results, the answering times seemed to be almost the same. In order to validate that the eWoT query-answering time was equivalent to that of GraphDB, a statistical significance test was performed. The well-known Iman–Davenport test [39] was applied to check if there was a statistically significant difference between GraphDB and eWoT answering times, using a confidence level of 95%.

The result of the Iman–Davenport test was a *p*-value of approximately 0.07. Since this value is above the established significance threshold (i.e., 0.05), it can be concluded that the query-answering time of eWoT was not significantly different from the GraphDB time. Therefore, it can be concluded from an efficiency point of view that the eWoT *Discovery* task was as efficient as a generic triple store such as GraphDB.

### 5.2. Experiment 2: Discovery, Distributed Access, and Translation

This experiment was aimed at testing the whole eWoT architecture, namely: *Discovery*, *Distributed Access*, and *Translation*. The goal of the experiment was to analyse the scalability of eWoT for an increasing number of IoT devices, and to analyse the overhead that the distributed access and translation introduce in the query-answering process.

For this purpose, a new set of 20 queries with different shapes was defined, namely: four linear queries, four star queries, four tree queries, and eight complex queries. The experiment consisted of running all the queries, ten times each, over GraphDB and over the SPARQL endpoint of eWoT. Every time a query was issued, three different times were stored: discovery time, access time plus translation, and the total answering time.

To carry out the experiment, a simulator of IoT devices was used. The simulator received as input the number of devices to be simulated and the registration endpoint of eWoT. Then, the simulator deployed that number of REST API endpoints, publishing different data in different http endpoints. Then, the simulator registered for each deployed REST API a Thing Description and its WoT-Mappings in eWoT. The Thing Descriptions registered were complete and complex, in contrast to those of Experiment 1, which were heterogeneous. The number of endpoints deployed for this experiment with the simulator were: 100, 250, 500, 750, and 1000.

**IoT devices simulator:** we implemented a simulator to simulate the different IoT devices that are discoverable and accessible in our ecosystem. The simulator took as input a CSV file containing the data of a smart house, extracted from the Machine Learning Repository of UC Irvine (https://archive.ics.uci.edu/ml/datasets/Individual+household+electric+power// +consumption), and the endpoint address of a description repository to register its TDs. When the simulator was started it registered itself into the repository (so our proposal could discover and access it), and also published a RESTful API whose data was updated each minute (following the CSV rows); in this way, the published data was the historical data of a smart house. Then, we developed a script that takes a number as input and starts as many simulators as specified in the input number; each in a different port.

**Figure 5:**
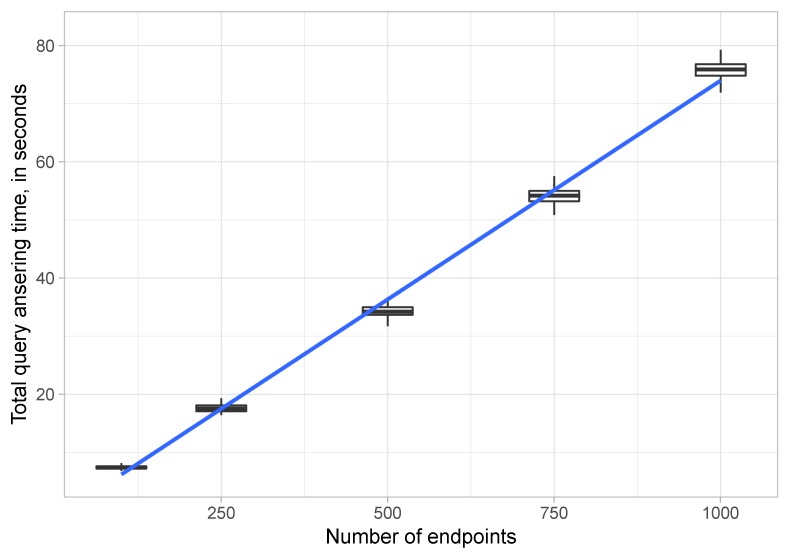
Averaged results: whiskers and plot of the results obtained handling different numbers of endpoints.

Figure 5 shows, relying on a whisker plot, the time taken to answer the different queries when different numbers of endpoints were available.

**Performance:** in light of the results depicted in Figure 5, several things can be concluded: (a) eWoT had a stable query answering time, that is, the response times were very similar (the boxes are very narrow); (b) eWoT took on average less than 20 s to solve queries that required accessing 240 endpoints, about 35 s to solve queries that required accessing 500, and 75 s for queries requiring that 1000 endpoints be accessed.

**Scalability:** the whisker plots of Figure 5 are not conclusive for establishing how eWoT scales. Therefore, a linear regression model [40] was applied with a confidence level of 95%. All the query- answering times shown in Figure 5 were used to feed the linear regression model. As a result, the query-answering times adjusted the model with a *p*-value less than $2.2\;\times \;10^{-16}$. In order to be a relevant result, its *p*-value should be below 0.05 due to the confidence level of 95%. Therefore, it can be concluded that eWoT scaled linearly since its results fit a linear regression model when the number of endpoints grew. This linear behaviour can be observed thanks to the blue line depicted in Figure 5, which corresponds to the regression line adjusted to the eWoT results.

**Distributed Access and Translation overhead:** Finally, as a last experiment we aimed at establishing how much overhead was introduced by the access regarding the discovery. Bear in mind that our experimental environment was located in the same machine, and thus the remote accesses were in our local host, meaning that the time to retrieve data tended to zero and the access time was only the time that our proposal took to fetch, translate the data into RDF, combine it with the discovery RDF data, and finally answer the query.

Table 3 reports the averaged percentage times that took answering the different queries, the table distinguishes the discovery and the access time percentages. Two main points should be noticed; the first is that the discovery and access percentages for all the different sizes of endpoints were almost the same (i.e., 96% for discovery and 4% for access), meaning that the linearity of our approach was preserved in both discovery and access. The second is that the discovery took most of the time whereas the access was nearly instant. This behaviour has two reasons: first, the network time in our experiment was almost zero, and second, the access is a task that can be parallelised whereas the discovery is not.

In light of the results shown in Table 3, we can conclude that the distributed access brings no overhead to the whole process of query answering, considering that the time to retrieve through the network tended to zero.

### 5.3. Experiment 3: eWoT vs. Centralised Approach

This experiment was aimed at comparing the query-answering time of eWoT and a centralised proposal, the integration efforts required by both, and how they behave. The goal of the experiment was to analyse the pros and cons of the eWoT approach and the well-known centralised approach from the literature.

For this purpose, a new set of 20 queries with different shapes was defined, namely: four linear queries, four star queries, four tree queries, and eight complex queries. The experiment consisted of issuing all the queries, ten times each, over the centralised proposal and eWoT. Every time a query was issued, the query-answering time was kept for further analysis.

Due to the lack of availability of centralised proposals in the literature, or due to hard-restrictions that made the use of some proposals unfeasible, we developed a custom proposal for the purposes of this experiment. The proposal was developed following the centralised approach reported by Zhou et al. [9].

The centralised proposal consisted of a GraphDB that stores the TDs of the IoT devices, and several services that monitor the API of the IoT devices being integrated. These services periodically pull data from the IoT devices, transform such data into RDF, and correctly inject the values to the corresponding TDs. In order to carry out this experiment the IoT devices involved were the ones published by the Stars4ALL European project. This project publishes in a REST API the real-time data of a large number of photometers distributed across the world (https://github.com/STARS4ALL).

**Figure 6:**
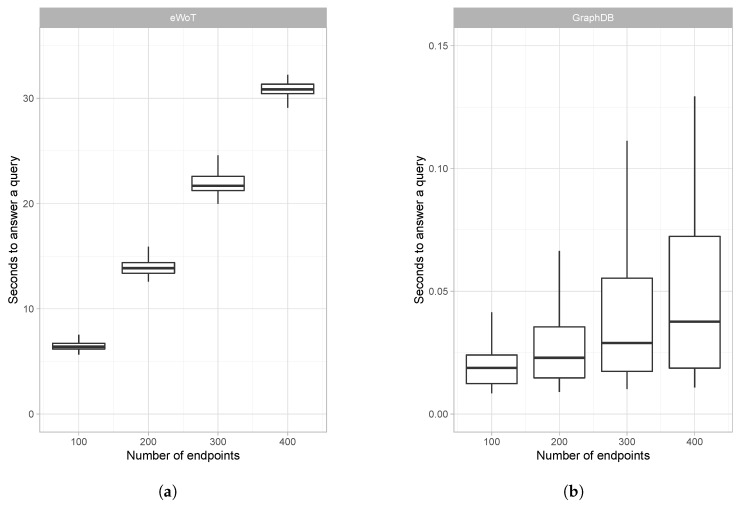
Comparison of eWoT and centralised approach based on GraphDB: (**a**) eWoT query-answering time and (**b**) GraphDB query answering time.

Figure 6 shows, using a whisker plot, the time taken to answer the different queries. Figure 6a reports the time required by eWoT, whereas Figure 6b reports the time required by GraphDB. Bear in mind that the charts have different scales in their *y*-axes.

**Performance:** observing the results reported by Figure 6, the centralised proposal based on GraphDB clearly outperformed the query-answering time of eWoT. This is because when a query was issued in this proposal, since the IoT data were already present in the triple store, the query was directly answered using the GraphDB SPARQL engine. In contrast, when a query was issued to eWoT, first a discovery task was performed and then all the IoT devices relevant to answer such query were accessed, their data retrieved and translated to RDF, and finally the query answer was computed.

**Data freshness:** the values reported by the IoT devices change constantly. Since the queries issued in this experiment involved such values, answering them with the latest reported values is paramount. eWoT retrieves their values anytime a query is issued, and therefore the freshness of data is guaranteed. In contrast, the centralised proposal relies on several services that monitor the IoT devices and periodically inject their values into GraphDB. As a result, the freshness of the IoT devices’ data cannot be guaranteed.

**Integration effort:** overcoming IoT devices’ heterogeneity and integrating new ones is a challenging task. eWoT address this task by requiring the registration of TDs containing WoT-Mappings any time a new IoT device must be integrated. This entails code development, providing users with a plug-and-play system in which an IoT device becomes automatically interoperable just by providing the complete TD. The centralised proposal addresses this task by requiring a practitioner to develop a service that monitors the IoT device and periodically uploads its value into the triple store.

In light of the results reported in Figure 6 and the previous analysis, several conclusions can be reached: (a) the centralised proposal was faster than eWoT answering the queries, but (b) the centralised proposal cannot guarantee that such queries are answered using the latest values of the IoT devices. Instead, eWoT always guarantees that the queries are answered with their latest values. (c) In addition, integrating new IoT devices in the centralised proposal requires the development of code, or code modification, whereas eWoT only requires the registration of a TD.

## 6. Conclusions

The latest efforts addressing IoT semantic interoperability issues have evolved towards the resolution of SPARQL queries in a transparent way in order to consume data from a large number of IoT devices, which may publish their data in RDF or rely on annotations to translate their data into RDF.

In this paper, we presented a theoretical proposal that is able to cope with IoT devices that does not necessarily rely on semantic Web technologies, and for a given query is able to produce an answer that may involve accessing such devices and translating their data into RDF when needed. Therefore, our proposal answers SPARQL queries involving a set of IoT devices transparently. In addition, we introduced a specific implementation of our proposal, for which we have proven that it (a) provides complete and correct answers with no statistical differences in the response time compared to a regular triple store; and (b) offers linear scalability for elaborating answers for queries that require discovery and access when coping with large ecosystems of IoT devices.

While the work presented in this paper establishes ground results towards semantic interoperability, there are some aspects which are not handled in the current implementation that are left for future lines of work.

The first issue is to enhance our mappings to extend the features supported, for example: allowing the definition of URI patterns, allowing the translation from more data formats, and supporting cleaning and transformation functions. In this sense, we plan to evolve the ontology network by including and adapting other mapping languages, such as RML [36].

The second issue to be addressed in future work relates to how privacy is handled. One approach consists of storing all the descriptions in one centralised repository to which all the clients are connected and their discovery and access mechanisms are filtered by Access Control List (ACL) policies. The main drawback of this solution is that the owner of the repository, which might be a private corporation, would control all the data. An alternative approach would rely on a decentralised deployment in which each client would host their own description repository. In this scenario, the clients would control with whom they exchange descriptions in order to allow others to discover and access their devices. The main drawback of this approach is that there is no trustworthy way to ensure that a third party client deletes a description when requested. To address this issue, blockchain transactions could be applied as legal contracts among clients. A further factor to be considered by these approaches relates to the potential vulnerabilities that IoT devices may expose [41]. 

## Figures and Tables

**Table 1:** Literature comparison.

	Semantic Interoperability		Zhou et al.	Solves
	IoT Device	IoT Ecosystem	Classification	SPARQL Query
Proposals [16,17,18,19,20,21,22,23,24,25,26,27,28]	✓	✗	-	✗
Guinard et al. [29]	✗	✓	Text Indexing	✗
Wei and Jin [30]	✗	✓	Centralised	✗
Cassar et al. [31]	✗	✓	Centralised	✓
Gyrard and Serrano [8,34]	✗	✓	Federation	✓
Zarko et al. [35]	✗	✓	Federation	✓
eWoT	✗	✓	Decentralised	✓

**Table 2:** Effectiveness: averaged results obtained by a triple store and our client plus the statistical significance of their differences.

	GraphDB		eWoT	
Query	Answer Size	Avg. Time (s)	Answer Size	Avg. Time (s)
Linear 1	1520	0.08	1520	0.13
Linear 2	8832	0.09	8832	0.10
Linear 3	9120	0.10	9120	0.11
Linear 4	9120	0.16	9120	0.17
Star 1	4566	0.11	4566	0.11
Star 2	3603	0.06	3603	0.06
Star 3	5202	0.07	5202	0.07
Star 4	800,000	24.78	800,000	25.78
Tree 1	39,258	0.48	39,258	0.57
Tree 2	506,529	10.23	506,529	9.24
Tree 3	506,529	15.48	506,529	20.98
Tree 4	800,000	35.4	800,000	37.89
Cycle 1	259	0.02	259	0.02
Cycle 2	1150	0.10	1150	0.09
Complex 1	852,042	25.68	852,042	28.73
Complex 2	317,040	7.15	317,040	13.89
Complex 3	217,500	11.80	217,500	11.32
Complex 4	215,700	6.46	215,700	10.84
Complex 5	215,700	11.20	215,700	10.32
Complex 6	215,700	12.12	215,700	15.23

**Table 3:** Percentage of Discovery time and Distributed Access plus Translation time.

	Query Answering Time in %									
	100 Endpoints		250 Endpoints		500 Endpoints		750 Endpoints		1000 Endpoints	
Query Type	Discovery	Access	Discovery	Access	Discovery	Access	Discovery	Access	Discovery	Access
Linear 1	95%	5%	96%	4%	96%	4%	96%	4%	95%	5%
Linear 2	96%	4%	96%	4%	96%	4%	96%	4%	96%	4%
Linear 3	95%	5%	96%	4%	96%	4%	96%	4%	96%	4%
Linear 4	95%	5%	96%	4%	96%	4%	96%	4%	96%	4%
Star 1	96%	4%	96%	4%	96%	4%	96%	4%	96%	4%
Star 2	96%	4%	96%	4%	96%	4%	96%	4%	96%	4%
Star 3	96%	4%	96%	4%	96%	4%	96%	4%	96%	4%
Star 4	96%	4%	96%	4%	96%	4%	96%	4%	96%	4%
Tree 1	96%	4%	96%	4%	96%	4%	96%	4%	96%	4%
Tree 2	95%	5%	96%	4%	96%	4%	96%	4%	96%	4%
Tree 3	96%	4%	96%	4%	96%	4%	96%	4%	96%	4%
Tree 4	96%	4%	96%	4%	96%	4%	96%	4%	96%	4%
Complex 1	93%	7%	94%	6%	96%	4%	95%	5%	96%	4%
Complex 2	95%	5%	96%	4%	96%	4%	96%	4%	96%	4%
Complex 3	95%	5%	96%	4%	96%	4%	96%	4%	96%	4%
Complex 4	95%	5%	96%	4%	96%	4%	96%	4%	96%	4%
Complex 5	95%	5%	96%	4%	96%	4%	96%	4%	96%	4%
Complex 6	95%	5%	96%	4%	96%	4%	96%	4%	96%	4%
Complex 7	96%	4%	96%	4%	96%	4%	96%	4%	96%	4%
Complex 8	95%	5%	96%	4%	96%	4%	96%	4%	96%	4%

## Abbreviations

The following abbreviations are used in this manuscript:

W3C	World Wide Web Consortium
WoT	Web of Things
TD	Web of Things Thing Description ontology
OP	Object Property
WoT-M	Web of Things Mappings ontology
O&M	Observation and Measures
RDF	Resource Description Framework
TED	Thing Ecosystem Description
CSV	Comma Separated Values
URI	Unified Resource Identifier
IoT	Internet of Things

## References

[1] Wortmann F., Flüchter K. (2015). Internet of Things. Bus. Inf. Syst. Eng..

[2] Hassan M.M., Song B., Huh E.N. A framework of sensor-cloud integration opportunities and challenges. Proceedings of the 3rd international conference on Ubiquitous information management and communication.

[3] Middleton P., Tully J., Kjeldsen P. (2013). Forecast: The Internet of Things, Worldwide.

[4] Press G. (2014). Internet of Things by the Numbers: Market Estimates and Forecasts.

[5] Rawat D.B., Brecher C., Song H., Jeschke S. (2017). Industrial Internet of Things: Cybermanufacturing Systems.

[6] Initiative E.P. (2018). Advancing IoT Platforms Interoperability.

[7] Ouksel A.M., Sheth A. (1999). Semantic interoperability in global information systems. ACM SIGMOD Rec..

[8] Gyrard A., Serrano M. Connected smart cities: Interoperability with seg 3.0 for the Internet of Things. Proceedings of the 2016 30th International Conference on Advanced Information Networking and Applications Workshops (WAINA).

[9] Zhou Y., De S., Wang W., Moessner K. (2016). Search techniques for the web of things: A taxonomy and survey. Sensors.

[10] World Wide Web Consortium (2013). SPARQL 1.1 Overview.

[11] Kaebisch S., Kamiya T. (2017). Web of Things (WoT) Thing Description.

[12] Mathew S.S., Atif Y., Sheng Q.Z., Maamar Z. Web of things: Description, discovery and integration. Proceedings of the 2011 International conference on Internet of Things and 4th International Conference on Cyber, Physical and Social Computing.

[13] Buil-Aranda C., Arenas M., Corcho O. (2011). Semantics and optimization of the SPARQL 1.1 federation extension. Extended Semantic Web Conference.

[14] Kaebisch S., Anicic D. Thing description as enabler of semantic interoperability on the Web of Things. Proceedings of the IoT Semantic Interoperability Workshop.

[15] Serena F., Poveda-Villalón M., García-Castro R. Semantic discovery in the web of things. Proceedings of the International Conference on Web Engineering.

[16] Yang G., Xie L., Mäntysalo M., Zhou X., Pang Z., Da Xu L., Kao-Walter S., Chen Q., Zheng L.R. (2014). A health-IoT platform based on the integration of intelligent packaging, unobtrusive bio-sensor, and intelligent medicine box. IEEE Trans. Ind. Inf..

[17] Giaffreda R., Capra L., Antonelli F. A pragmatic approach to solving IoT interoperability and security problems in an eHealth context. Proceedings of the 2016 IEEE 3rd World Forum on Internet of Things (WF-IoT).

[18] Hosek J., Masek P., Andreev S., Galinina O., Ometov A., Kropfl F., Wiedermann W., Koucheryavy Y. (2017). A SyMPHOnY of integrated IoT businesses: closing the gap between availability and adoption. IEEE Commun. Mag..

[19] Datta S.K., Bonnet C., Nikaein N. An IoT gateway centric architecture to provide novel M2M services. Proceedings of the 2014 IEEE World Forum on Internet of Things (WF-IoT).

[20] Castellani A.P., Fossati T., Loreto S. HTTP-CoAP cross protocol proxy: an implementation viewpoint. Proceedings of the 2012 IEEE 9th International Conference on Mobile Ad-Hoc and Sensor Systems (MASS 2012).

[21] Al-Fuqaha A., Khreishah A., Guizani M., Rayes A., Mohammadi M. (2015). Toward better horizontal integration among IoT services. IEEE Commun. Mag..

[22] Bröring A., Schmid S., Schindhelm C.K., Khelil A., Käbisch S., Kramer D., Le Phuoc D., Mitic J., Anicic D., Teniente E. (2017). Enabling IoT ecosystems through platform interoperability. IEEE Softw..

[23] Li W., Privat G., Le Gall F. Towards a semantics extractor for interoperability of IoT platforms. Proceedings of the 2017 Global Internet of Things Summit (GIoTS).

[24] Li W., Privat G. Cross-Fertilizing Data through Web of Things APIs with JSON-LD. Proceedings of the European Semantic Web Conference.

[25] Jabbar S., Ullah F., Khalid S., Khan M., Han K. (2017). Semantic interoperability in heterogeneous IoT infrastructure for healthcare. Wirel. Commun. Mob. Comput..

[26] Yachir A., Djamaa B., Mecheti A., Amirat Y., Aissani M. (2016). A comprehensive semantic model for smart object description and request resolution in the Internet of Things. Procedia Comput. Sci..

[27] Soldatos J., Kefalakis N., Hauswirth M., Serrano M., Calbimonte J.P., Riahi M., Aberer K., Jayaraman P.P., Zaslavsky A., Žarko I.P. (2015). Openiot: Open source internet-of-things in the cloud. Interoperability and open-source solutions for the Internet of Things.

[28] Desai P., Sheth A., Anantharam P. Semantic gateway as a service architecture for iot interoperability. Proceedings of the 2015 IEEE International Conference on Mobile Services.

[29] Guinard D., Trifa V., Karnouskos S., Spiess P., Savio D. (2010). Interacting with the soa-based Internet of Things: Discovery, query, selection, and on-demand provisioning of web services. IEEE Trans. Serv. Comput..

[30] Wei Q., Jin Z. Service discovery for Internet of Things: A context-awareness perspective. Proceedings of the Fourth Asia-Pacific Symposium on Internetware.

[31] Cassar G., Barnaghi P., Wang W., Moessner K. A hybrid semantic matchmaker for iot services. Proceedings of the 2012 IEEE International Conference on Green Computing and Communications.

[32] Gomes P., Cavalcante E., Batista T., Taconet C., Chabridon S., Conan D., Delicato F.C., Pires P.F. (2016). A QoC-aware discovery service for the Internet of Things. Ubiquitous Computing and Ambient Intelligence.

[33] Gomes P., Cavalcante E., Batista T., Taconet C., Conan D., Chabridon S., Delicato F.C., Pires P.F. (2019). A semantic-based discovery service for the Internet of Things. J. Internet Serv. Appl..

[34] Gyrard A., Serrano M. A unified semantic engine for Internet of Things and smart cities: From sensor data to end-users applications. Proceedings of the 2015 IEEE International Conference on Data Science and Data Intensive Systems.

[35] Žarko I.P., Mueller S., Płociennik M., Rajtar T., Jacoby M., Pardi M., Insolvibile G., Glykantzis V., Antonić A., Kušek M. The symbIoTe Solution for Semantic and Syntactic Interoperability of Cloud-based IoT Platforms. Proceedings of the 2019 Global IoT Summit (GIoTS).

[36] Dimou A., Vander Sande M., Colpaert P., Verborgh R., Mannens E., Van de Walle R. (2014). RML: A Generic Language for Integrated RDF Mappings of Heterogeneous Data. LDOW.

[37] Stocker M., Seaborne A., Bernstein A., Kiefer C., Reynolds D. (2008). SPARQL Basic Graph Pattern Optimization Using Selectivity Estimation. Proceedings of the 17th International Conference on World Wide Web (WWW ’08).

[38] Wylot M., Hauswirth M., Cudré-Mauroux P., Sakr S. (2018). RDF data storage and query processing schemes: A survey. ACM Comput. Surv..

[39] Pereira D.G., Afonso A., Medeiros F.M. (2015). Overview of Friedman’s test and post-hoc analysis. Commun. Stat. Simul. C.

[40] Muggeo V.M. (2008). Segmented: An R package to fit regression models with broken-line relationships. R News.

[41] Gao J., Yang X., Jiang Y., Song H., Choo K.K.R., Sun J. (2019). Semantic Learning Based Cross-Platform Binary Vulnerability Search For IoT Devices. IEEE Trans. Ind. Inf..

